# Assessment of the Susceptibility to Material Fracture in the Cross-Wedge Rolling Process with Concave Tools

**DOI:** 10.3390/ma15196605

**Published:** 2022-09-23

**Authors:** Tomasz Bulzak

**Affiliations:** Mechanical Engineering Faculty, Lublin University of Technology, 36 Nadbystrzycka Str., 20-618 Lublin, Poland; t.bulzak@pollub.pl; Tel.: +48-81-538-424

**Keywords:** cross-wedge rolling, concave tools, damage, fracture, fem

## Abstract

The internal cracking of forgings during the cross-wedge rolling (CWR) process is a serious limitation that prevents the correct implementation of this process. The phenomenon of material cracking in the CWR process reduces the technological and application possibilities of this highly efficient process, which can produce forgings with high geometric accuracy. This article presents the results of rolling forgings at different temperatures. An analysis of the results showed that the size of the resulting material fracture in the CWR process is related to the size of the ovalisation of the cross-section of the forging formed during rolling. On the basis of the observations made, it was proposed to realise the cross-wedge rolling process with concave tools. The use of tools with a concave geometry is intended to reduce the excessive flow of material in the rolling direction, which restrains the formation of the ovalisation of the cross-section of the forging. Numerical simulations were carried out comparing the rolling with flat tools and concave tools with different radii of the curvature. The results show that the use of concave tools reduces the ovality of the cross-section of the forging during rolling and reduces the value of the normalised Cockcroft–Latham (CL) fracture criterion.

## 1. Introduction

Cross-wedge rolling (CWR) is a technology used to produce axisymmetric products. Cross-wedge rolling is still being developed, and newer technologies based on this rolling method are being developed. Recent work published in the last 20 years on cross-wedge rolling technology has focused on: material fractures in the CWR process, the microstructures of rolled products, the rolling of hollow products, waste minimisation in the CWR process, the rolling of hybrid materials and the general influence of parameters on the rolling process.

Recent studies on the problem of cracking in the CWR process have provided three fracture criteria for predicting cracking in this process. The first hybrid criterion was presented by Pater et al. [1]. The second stress criterion was presented by Zhou et al. [2]. In both cases, tests were presented to determine the critical values of the presented fracture criteria. Another fracture criterion was presented by Yamane et al. [3]. Ghiotti and Novella [4] conducted a study on the adaptation of available fracture criteria for crack prediction in the cross-wedge rolling process.

Pater et al. [5] conducted an evaluation of 10 ductile fracture criteria in the context of the quality of crack prediction in the CWR process. The study showed that, in addition to the fracture criterion itself, it is very important to know the limit values of the fracture criteria. Pater et al. [6] also showed that for crack prediction in the cross-wedge rolling process, criteria with a stress dimension are more suitable. Bulzak [7] investigated nine ductile fracture criteria for the quality of crack prediction in two variants of the CWR process, i.e., conventional rolling and multi-wedge rolling. The results indicate that each rolling method requires a different fracture criterion for correct crack prediction. Zhou et al. [8] analysed four fracture criteria for 27 rolling cases with varying geometrical process parameters. The results obtained did not confirm the suitability of any of the criteria used. Furthermore, it was observed that material cracking occurred at different values of the fracture criteria.

The microstructural changes in forgings produced by warm and hot cross-wedge rolling were analysed by Bulzak et al. [9]. The warm cross-wedge rolling process was also studied by Huang et al. [10]. Xiong et al. [11] studied the microstructural changes and strength properties of warm cross-wedge-rolled high-carbon steel bars. Zhang et al. [12] also discussed the problem of microstructural changes during cross-wedge rolling in their study. Yuan et al. [13] analysed the microstructural changes in Ti-6Al-4V titanium alloy forgings after the cross-wedge rolling process.

The engineering industry, at a time when there is a very strong emphasis on reducing the weights of structures, frequently uses hollow products, including axles and shafts. In this context, research work is also being undertaken that focuses on the problem of the cross-wedge rolling of hollow products. One of the first papers dealing with the issue of the cross-wedge rolling of hollow products was published by Bartnicki and Pater [14]. Developments in the rolling of hollow products have led to the use of mandrels to stabilise and calibrate the hole of the rolled shaft or axle. The issue of the use of a mandrel in the cross-wedge rolling process has been addressed, among others, by Shen et al. [15]. In the course of conducting research, Bartnicki et al. [16] proved that the best conditions for forming hollow products in the cross-wedge rolling process occur when three rolling tools are used. Based on the assumption that hollow products should be rolled with three rollers, a rotary compression process was developed and analysed by Tomczak et al. [17].

In recent times, attempts have also been made to produce bimetallic materials using cross-wedge rolling technologies. Wu et al. [18] presented the results of a study on the joining of two steel grades using a cross-wedge rolling process. Kruse et al. [19] showed that the microstructure at the interface between two steels is significantly more favourable when the materials are joined by the cross-wedge rolling process than by conventional welding. Highly successful results in joining different grades of steel were obtained by Tomczak et al. [20] using a three-roll skew rolling system.

Minimising material consumption in cross-wedge rolling processes is also a very important aspect. The minimisation of the sizes of end cavities by selecting appropriate process implementation parameters was proposed by Pater et al. [21]. Research in this area was also conducted by Han et al. [22], who used a billet with tapered ends for cross-wedge rolling.

From the literature review presented above, it can be seen that the research issues related to the cross-wedge rolling process are very extensive. However, the main problem of cross-wedge rolling technology is internal cracking, which, despite the many advantages of CWR, may disqualify this manufacturing technology in favour of ones that are less attractive in terms of productivity, energy and material consumption but guarantee products that are free of internal defects. 

The research in this article aimed to identify the causes of the increase in the damage function value during cross-wedge rolling. Tests were carried out at different temperatures in the cross-wedge rolling process, resulting in forgings with different crack sizes. Using FE simulations, it was determined that the increase in damage values is due to an increase in plastic strain, which results from the excessive ovalisation of the forging cross-section during rolling. Knowing the reasons for the increase in damage values, a new method of cross-wedge rolling was proposed, using concave tools to minimise the value of the damage function by reducing ovalisation, which translates into lower values of plastic strain. In addition, the influence of the size of the tool concavity, determined by the value of the radius of the curvature of the tools, on the propensity of the material to fracture during the CWR process was also analysed. Thus, new knowledge is provided on the influence of the main parameter describing the geometry of new tools on the propensity of the material to fracture.

## 2. Materials and Methods

### 2.1. Experimental Studies of the Cross-Wedge Rolling Process 

**Figure 1:**
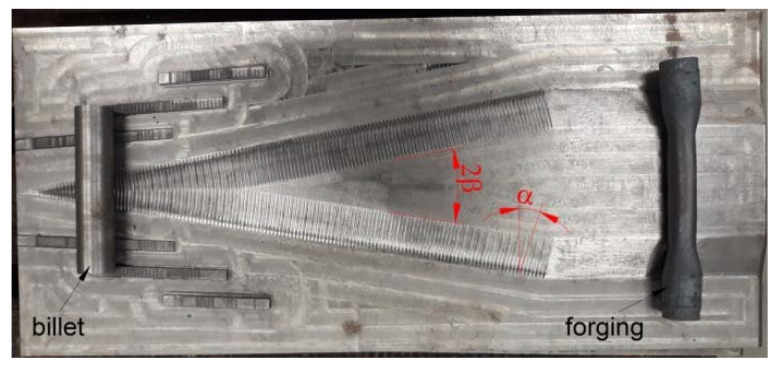
The wedge tool used in the experimental study.

The cross-wedge rolling process for forgings was carried out under laboratory conditions using flat tools, as shown in Figure 1. The geometry of the wedge tools was described by two angle parameters: forming angle *α* = 15° and spreading angle *β* = 10°. A flat-rolling mill with one moving tool was used for rolling tests. The moving tool moved at a constant velocity of 300 mm/s during rolling. Rolling tests were carried out at three initial billet temperatures, i.e., 950 °C, 1050 °C and 1150 °C. The billet material was heated in an electric chamber furnace before rolling. The billet material was C45-grade steel. The billet for the rolling process was a bar with dimensions of *D* = 33 mm and *L* = 160 mm. During the rolling process, a constriction with a diameter of *d* = 22 and *l* = 100 mm was rolled out on the billet bar. 

### 2.2. Finite Element Analysis of the Cross-Wedge Rolling Process

Numerical modelling was carried out to identify the causes of crack formation during the cross-wedge rolling of forgings. Numerical simulations were performed in Simufact Forming (v.15, MSC Software Company, Hamburg, Germany) software. The numerical simulation used identical conditions to those adopted in the experimental studies. A rigid-plastic von Mises material with isotropic hardening was used to model a billet. The rheology of C45 steel is described by the following equation:

(1) $$\sigma_{F}=1521.3\cdot e^{-0.0027T}\cdot \varepsilon^{-0.1265}\cdot e^{-0.05958/\varepsilon }\cdot {\dot{\varepsilon }}^{0.1454}$$

where *σ_F_* is the flow stress, *ε* is the effective strain, $\dot{\varepsilon }$ is the effective strain rate, and *T* is the temperature. The constitutive equation (Equation (1)) adopted in this study to describe the rheology of C45 steel has been used successfully on several occasions in studies conducted by other authors [23,24,25]. For the discretisation of the billet, hexahedral finite elements with an average size of 1.5 mm were used. The contact conditions were described by a Tresca friction model, for which a friction factor of *m* = 0.8 was assumed [26,27]. Heat transfer during rolling was described using a heat transfer coefficient between the billet and tools of 20 kW/m^2^K and a heat transfer coefficient between the billet and ambient of 50 W/m^2^K. It was also assumed that 90% of the plastic deformation work and frictional work is converted into heat.

### 2.3. Validation of the Numerical Model 

**Figure 2:**
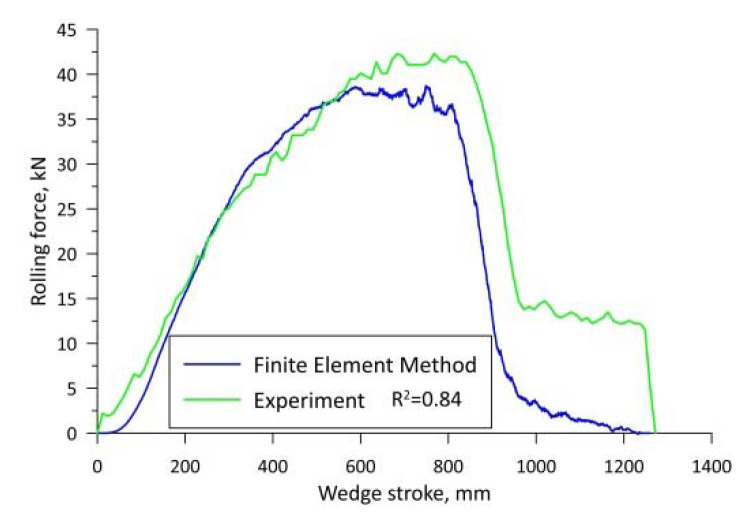
Summary of rolling forces obtained during the experiment and determined by the finite element method.

Validation of the developed numerical model was based on a comparison of the force characteristics of the rolling process measured during the experiment and those obtained during numerical simulations. Validation was performed for the rolling case at 1150 °C. This temperature was selected because it produced the least interference from material cracking. To date, observations of the cross-wedge rolling process have shown that at lower rolling temperatures, when the cracks are of larger size on the rolled step, a very high ovality of the cross-section is formed. The large ovality of the cross-section of the forging results in an intense increase in the rolling force during the experiment. On the other hand, during numerical simulations, the rolled material is a continuous medium and does not separate, as a result of which the ovality of the cross-section of the forging is smaller, and thus, the rolling forces are lower. Therefore, the validation of the numerical model at lower rolling realisation temperatures, especially when a fracture occurs, is subject to large errors due to the fracture of the rolled material. Figure 2 presents the force characteristics obtained from the experiment and the numerical modelling. The coefficient of determination *R*^2^ for these two graphs is 0.84, confirming the high agreement between the numerical model and the experimental process. In the first phase of the process, the correspondence between the force plots is very high. The discrepancy in results starts when the wedge has moved 600 mm. This is most likely to be the point at which a fracture develops. The resulting crack and its growth intensify the ovalisation of the forging cross-section, which generates higher rolling forces during the experiment. 

## 3. Results

**Figure 3:**
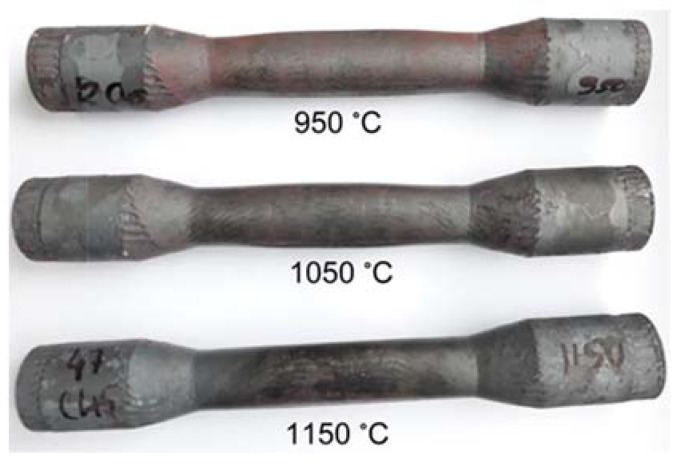
Forgings obtained in experimental tests.

The experimental tests produced the forgings shown in Figure 3. The forgings have no visible external defects. In the central part of the rolled step, a pronounced ovalisation of the cross-section is observed, which decreases with increasing process realisation temperature. The ovalisation observed may indicate that cracks have formed in the centre of the forgings. The sizes of the resulting ovalisation were measured and are summarised in Table 1. The measurement results obtained clearly show that the size of the ovalisation of the cross-section increases as the process implementation temperature decreases. 

**Figure 4:**
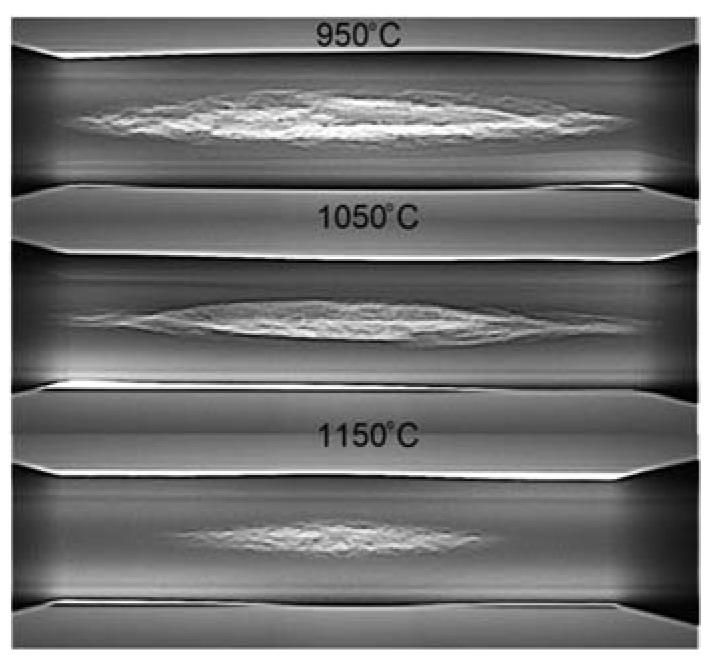
Internal cracks in the obtained forgings as shown in the X-ray radiograph.

Figure 4 shows X-ray radiographs of the central steps of the obtained forgings. The radiographs obtained confirm that a crack has formed in the central part of each of the forgings obtained. The size of the created fracture depends on the temperature at which the process is carried out. An increase in the process realisation temperature leads to a reduction in the size of the fracture created, which also translates into a smaller ovalisation size of the cross-section of the step in which the fracture was created. 

**Figure 5:**
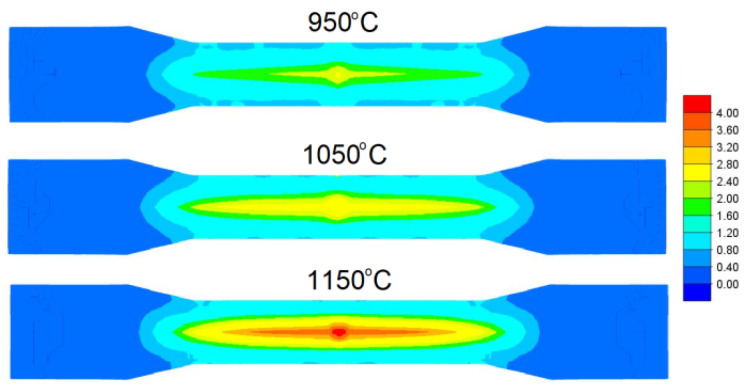
Distribution of the normalised Cockcroft–Latham damage criterion for different temperatures.

Figure 5 shows the distribution of the normalised Cockcroft–Latham (CL) damage criterion. This criterion has the following mathematical form:

(2) $$f_{nCL}=\int_{0}^{\varepsilon }\frac{\sigma_{1}}{\sigma_{i}}d\varepsilon ,$$

where *f_nCL_* is the ductile damage criterion value, *ε* is the effective strain, *σ*_1_ is the maximum principal stress, and *σ_i_* is the effective stress.

The damage criterion distribution obtained shows that the highest damage criterion values were obtained when rolling at 1150 °C. In contrast, the smallest values of the damage criterion were obtained when rolling at 950 °C. The damage criterion distribution shown is not consistent with the results shown in Figure 4, where the largest recorded crack size is at 950 °C. However, in this case, the limit value of the damage criterion at different temperatures of the rolling process realisation must be taken into account. Pater et al. [28] proved that the limit value of the damage criterion is temperature dependent. In this case, the size of the crack can be estimated by comparing percentage by which the damage criterion values exceed the limit value of the individual process realisation temperatures. Pater et al. [28], on the basis of their study, reported that the Cockcroft–Latham damage criterion limits for the C45 steel grade are: 0.84 for 950 °C, 1.76 for 1050 °C and 3.72 for 1150 °C.

**Figure 6:**
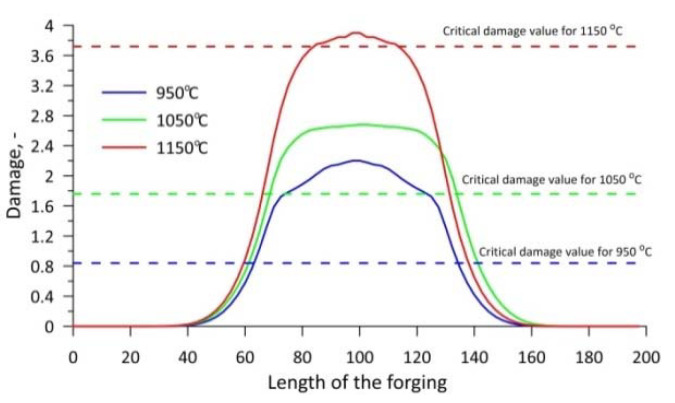
Distribution of damage values along the axis of the forgings and critical damage values.

Figure 6 shows a graph in which the damage criterion value along the central axis of the individual forgings and the fracture criterion limit values for the individual temperatures are presented in numerical form. The presented graph confirms that as the temperature increases, the damage criterion value and the fracture limit value increase, beyond which the material fractures increase. It can be seen from the data presented that the damage limit values were exceeded at all rolling temperatures. The lower the rolling temperature, the greater the exceedance of the limit value. In the cases analysed, the limits were exceeded by 161.9%, 52.27% and 4.84% at 950 °C, 1050 °C and 1150 °C, respectively. The percentage by which the damage limit value was exceeded is reflected in the size of the cracks shown in Figure 4. From the results obtained, it can therefore be concluded that, from the point of view of material fracture in the CWR process, not only the damage criterion value but also the damage limit value at a given temperature is important.

The results outlined above indicate that the damage value is strongly dependent on the rolling temperature. It is therefore worth identifying the reason for the lower damage values at lower rolling temperatures and the possibility of reducing the damage values at higher temperatures. Reducing the damage value at higher rolling temperatures while the limits for these temperatures are quite high would provide tangible benefits in terms of minimising the probability of material fracture in CWR processes.

## 4. Discussion

The value of the Cockcroft–Latham damage criterion used in the analysis is dependent on the state of stress and strain occurring during the cross-wedge rolling process. It was therefore decided to analyse how the individual components influence the damage value changes during the process.

### 4.1. Stress-State Indicator σ_1_/σ_i_

**Figure 7:**
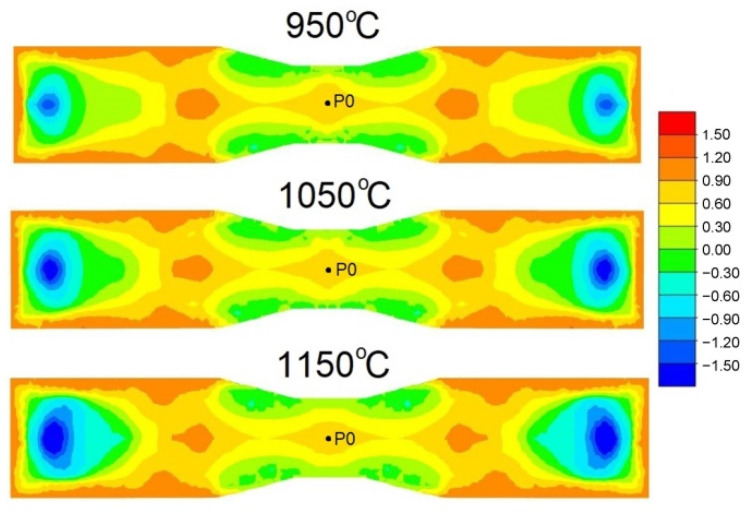
Distribution of the stress-state indicator values *σ*_1_*/σ_i_*.

The sub-integral function of the CL damage criterion is dependent on the ratio of the maximum principal stress *σ*_1_ to the effective stress *σ_i_*. Therefore, it was decided to analyse how the value of the indicator *σ*_1_*/σ_i_* changes during rolling at different temperatures. Figure 7 shows, in the form of a colour map, the distribution of the indicator values *σ*_1_*/σ_i_* at different rolling temperatures. In all cases, the distribution of the indicator *σ*_1_*/σ_i_* is shown at the same forging position along the tool length. The distribution shows that, irrespective of the rolling temperature, the indicator *σ*_1_*/σ_i_* assumes identical values in the range of 0.6/0.9 in the central part of the forming zone. A modification of the rolling temperature results in proportional changes in the maximum principal stress and effective stress during rolling. Consequently, the indicator *σ*_1_*/σ_i_* takes on similar values independently of the rolling temperature.

**Figure 8:**
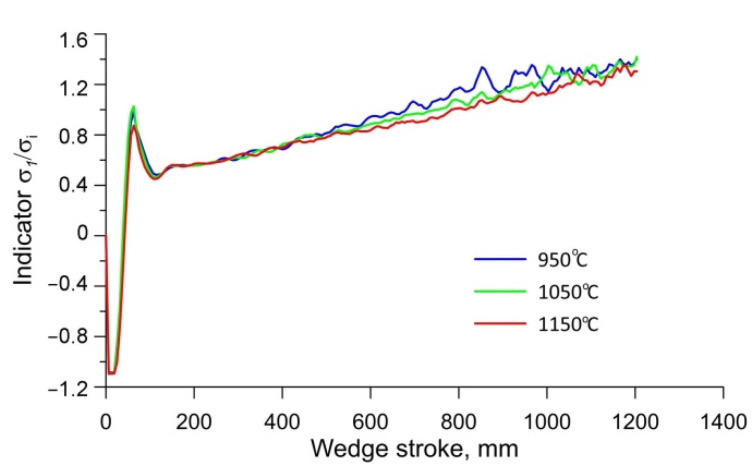
Variation in the stress-state indicator *σ*_1_*/σ_i_* at point P0 at different rolling temperatures.

Figure 7 shows that the distribution of the stress-state indicator *σ*_1_*/σ_i_* is almost identical at a given moment in time, which would indicate that the rolling temperature has no influence on the indicator *σ*_1_*/σ_i_*. In order to further analyse the changes in the stress-state indicator *σ*_1_*/σ_i_*, it was decided to analyse the changes in this parameter throughout the process. For this purpose, the changes in the stress-state indicator *σ*_1_*/σ_i_* were analysed at point P0, which was located in the centre of the forging, as shown in Figure 7. The location of point P0 was deliberately chosen in the axis of the forging, as this is the point where crack initiation occurs during cross-wedge rolling. The changes in the stress-state indicator *σ*_1_*/σ_i_* are illustrated in Figure 8. It can be seen from the graph that the course of changes in the indicator *σ*_1_*/σ_i_* values at all rolling temperatures is identical. At the initial stage of rolling, when the material is gripped by the tools, the indicator *σ*_1_*/σ_i_* value oscillates from −1.0 to 1.0, and then the indicator *σ*_1_*/σ_i_* value stabilises at around 0.6. A linear increase in the value of the indicator *σ*_1_*/σ_i_* can be observed in a further part of the process. At a wedge displacement of 400 mm, a slight increase in the indicator *σ*_1_*/σ_i_* can be observed as the initial rolling temperature decreases. The slightly higher values of the indicator *σ*_1_*/σ_i_* at a rolling temperature of 950 °C do not explain the lower values of the damage criterion at this rolling temperature (Figure 5). The presented waveforms of the stress-state indicator *σ*_1_*/σ_i_* indicate the opposite situation, namely, that this parameter will cause the damage criterion value to increase with decreasing rolling temperature.

In conclusion, it can be said that the stress-state indicator *σ*_1_*/σ_i_* at different rolling temperatures has a negligible effect on the value of the damage criterion. Consequently, the differences in the damage criterion value should be attributed to the strain state.

### 4.2. Effective Strain ε_i_

**Figure 9:**
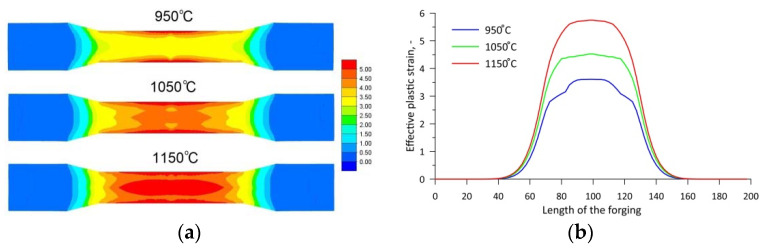
Effective plastic strain values at different rolling temperatures: (**a**) distribution map in the axial section; (**b**) distribution along the central axis of the forgings.

Figure 9 shows maps with the distribution of the effective strain at individual rolling temperatures. The distributions show that the rolling temperature has a very significant effect on the distribution and value of effective strain. By far, the lowest values of effective strain occur when rolling at 950 °C. An increase in the temperature results in an increase in effective strain values. In all rolling cases, very high effective strain values can be observed in the near-surface region of the middle step of the forging. In the central part of the forging, the effective strains are 3.5, 4.5 and 5 at temperatures of 950 °C, 1050 °C and 1150 °C, respectively.

**Figure 10:**
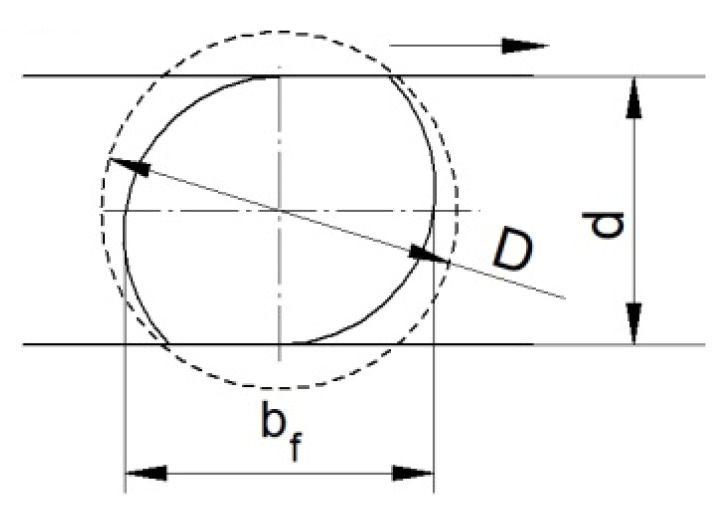
Schematic of the CWR process with flat tools with marked geometrical parameters.

The effective strain distributions shown indicate that the damage criterion values are strongly dependent on this parameter. It is therefore reasonable to determine the reason for this. The increase in effective strain values at higher temperatures is probably due to the occurrence of significant redundant strain. Redundant strain can arise from the excessive ovalisation of the cross-section of the forging during rolling. The high ductility of the material at higher rolling temperatures may favour the excessive ovalisation of the cross-section. The excessive ovalisation of the cross-section will result in the occurrence of an increased intermediate deformation ratio, which will generate additional plastic strain. Figure 10 shows a schematic representation of the cross-sectional rolling process, together with designations from which the theoretical deformation ratio and intermediate deformation ratio can be described. The theoretical deformation ratio *δ_t_* is expressed as the ratio of the billet diameter *D* to the forging diameter *d*:

(3) $$\delta_{t}=\frac{D}{d}.\;$$

In contrast, the intermediate (secondary) deformation ratio *δ_i_* will depend on the size of the ovalisation described by the width of the oval *b_f_* and the diameter of the forging *d*:

(4) $$\delta_{i}=\frac{b_{f}}{d}.\;$$

It follows from Relation (4) that the greater the ovalisation during rolling, the greater the intermediate deformation ratio *δ_i_*, which, with high ovalisation values, can result in higher values of redundant strain. It is therefore useful to analyse how the degree of the ovalisation of the cross-section looks during rolling at different temperatures.

### 4.3. Ovalisation of the Cross-Section of the Forging

**Figure 11:**
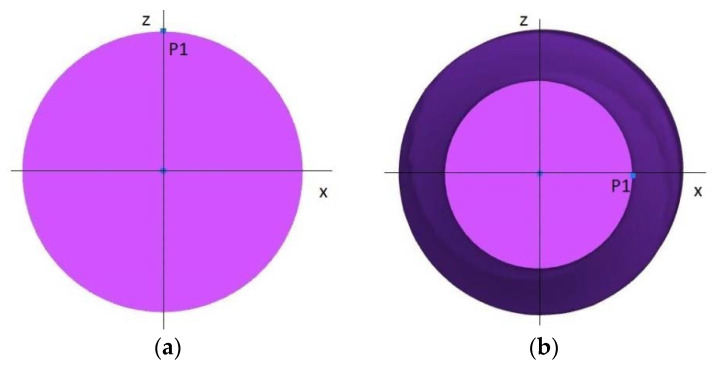
Location of point P1: (**a**) initial position on the surface of the billet; (**b**) final position on the surface of the forging.

In order to determine the degree of ovalisation during rolling at different temperatures, point P1 was extracted from the surface of the billet, as shown in Figure 11. The trajectory of the movement of the selected point P1 during rolling was then determined.

**Figure 12:**
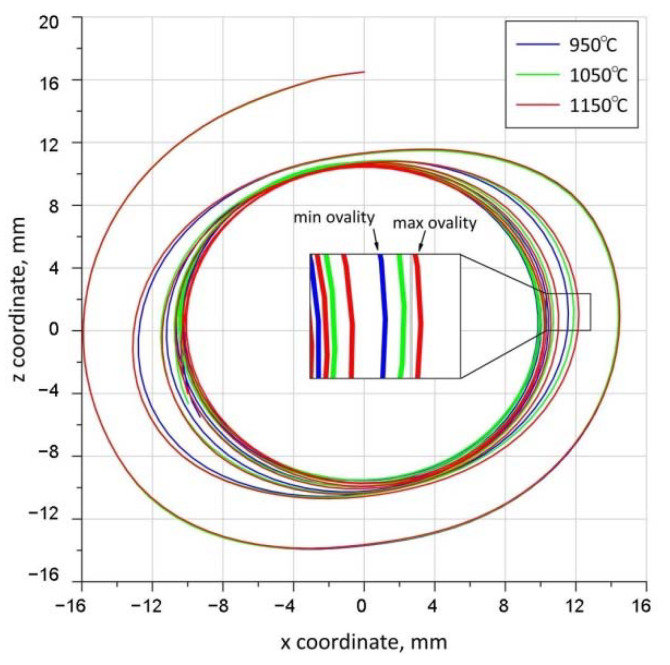
Trajectories of movement of point P1 at different rolling temperatures.

The obtained trajectories of the movement of point P1 during rolling at the adopted temperatures are shown in Figure 12. The trajectories of the movement of point P1 indicate that the highest ovality of the cross-section during rolling occurs during rolling at 1150 °C, while the lowest occurs during rolling at 950 °C. The increase in the ovalisation of the cross-section when rolling at higher temperatures is a result of the greater ductility of the material. The more ductile material undergoes greater elongation in the rolling direction as a result of the wedges, which increases the width *b_f_* of the resulting oval. Therefore, when rolling at higher temperatures, an increase in the width of the oval leads to an increase in the value of the intermediate (secondary) deformation ratio *δ_i_*. An increase in the value of the intermediate deformation ratio *δ_i_* will result in an increase in the value of unnecessary plastic deformation. As a result, when rolling at higher temperatures, the calibration of the cross-section of the forging will be carried out over a longer forming path.

An analysis of the influence of the stress and strain states on the value of the Cockcroft–Latham damage criterion showed that, when rolling at different temperatures, the value of the effective strain has a greater influence on the damage criterion. However, the effective strain values shown by the analysis are dependent on the rolling temperature and the amount of ovalisation of the section that occurs during rolling. At this point, it is possible to formulate the hypothesis that a reduction in the ovalisation of the cross-section during cross-wedge rolling without changing the temperature value will result in a reduction in the value of the damage criterion.

## 5. Concept of Reducing the Cross-Section of Ovalisation during Cross-Wedge Rolling

**Figure 13:**
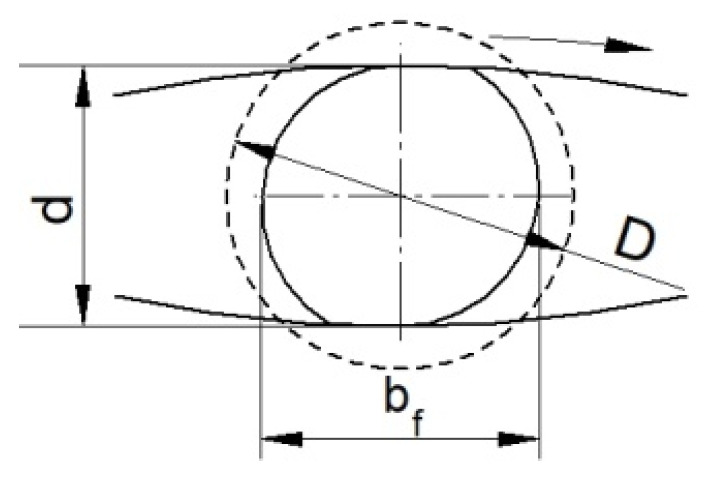
Scheme of the concave-wedge CWR process with marked geometrical parameters.

In order to achieve the minimum ovalisation of the cross-section of the forging during cross-wedge rolling without changing the process execution temperature, it is necessary to consider design changes to the tools or the kinematics of the CWR process execution. In the proposed solution, the tool geometry was modified, which also necessitated a change in the kinematics of the CWR process. In order to limit the ovalisation of the cross-section of the forging during CWR, it is proposed that the tools should girdle the forging during rolling, thus limiting excessive material flow in the rolling direction. The adopted CWR scheme with concave wedges is shown in Figure 13.

### 5.1. FEM Model of Cross-Wedge Rolling with Concave Tools

**Figure 14:**
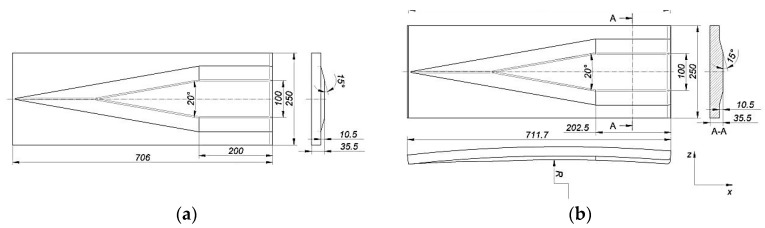
Geometry of the tools used for FEM analysis: (**a**) flat wedge; (**b**) concave wedge.

In order to prove the hypothesis formulated, numerical calculations were carried out for the cross-wedge rolling process with concave tools. Calculations were also carried out with the same process parameters using flat tools, and the results obtained were taken as a reference. To extend the research carried out, an analysis was also carried out to determine the effect of the size of the wedge concavity (radius *R*) on the value of the damage criterion. Figure 14 shows the geometry of the wedge tools used for the analysis. For both flat and concave tools, identical tool angle values (*α* = 15° and *β* = 10°) were used. The other tool dimensions were also selected so that the flat tool and the concave tool were as close to each other as possible. In the case of concave tools, tools with different degrees of concavity were used for the analysis, which was determined by the size of the radius *R*. The designed concave tools had curvatures with radii *R* = 2500 mm, 3750 mm, 5000 mm and 6250 mm.

**Figure 15:**
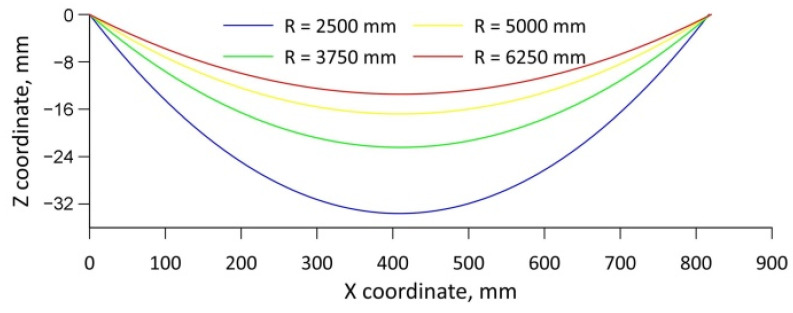
The trajectories of movement of wedge-shaped concave tools.

Numerical calculations were carried out using Simufact Forming software. It was assumed that the billet would be a bar of diameter *D* = 50 mm and length *L* = 142 mm made of C45-grade steel. The rheology of C45 steel is described by Equation (1). During the process, a reduction with a diameter of *d* = 35 and a length of *l* = 100 mm was rolled off the billet. In this variant of the numerical calculation, it was assumed that both tools performed the movement at a speed of 300 mm/s. For the flat tools, the tool path of motion was linear. The concave tools followed a curvilinear trajectory at a constant speed of 300 mm/s. The motion path of the concave tools was dependent on the value of the tool’s radius of curvature *R* and is shown in Figure 15. The adoption of two moving tools in this variant of the calculation facilitated the method of central positioning of the billet between the concave tools. The correct positioning of the charge ensures the elimination of the adverse phenomena of the advance and retardation of billet movement on the tools. The other boundary conditions adopted in the numerical simulations were identical to those described in Section 2.2.

### 5.2. Results of FEM Analysis

**Figure 16:**
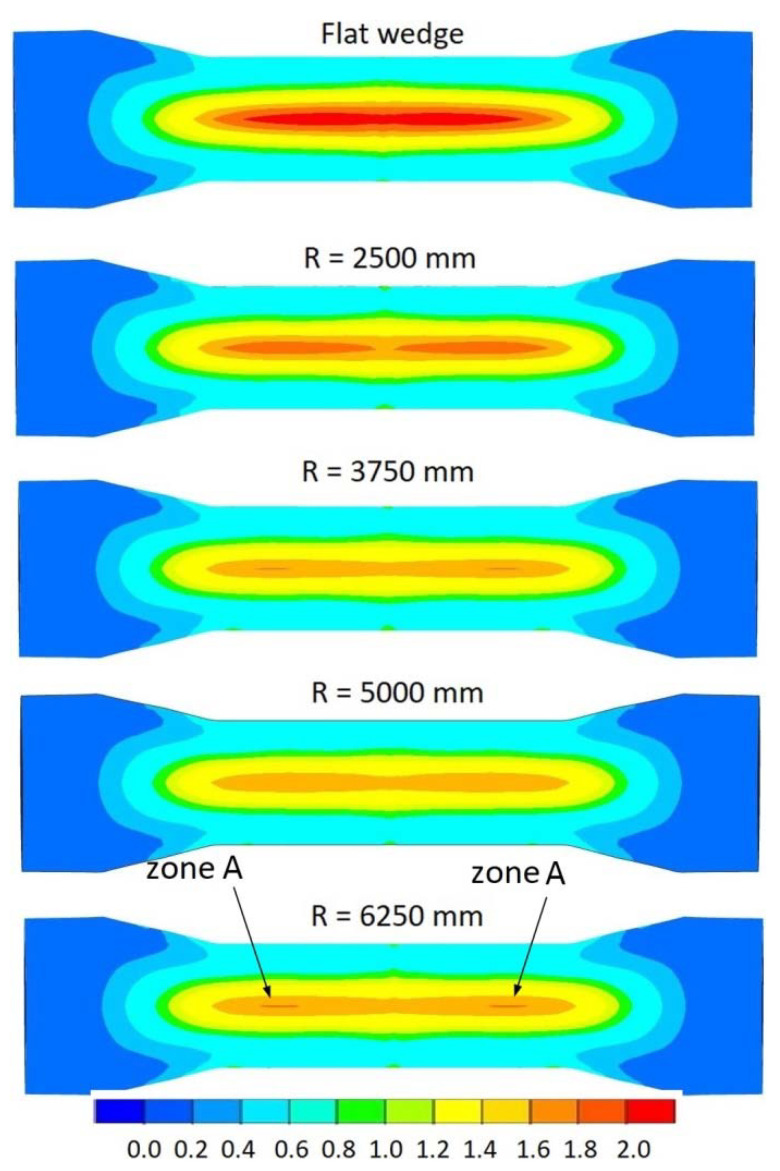
Distribution of the normalised Cockcroft–Latham damage criterion.

Figure 16 shows the distribution of the normalised Cockcroft–Latham (CL) damage criterion. The damage criterion for all analysed cases takes the largest values in the central zone of the forgings. The highest value of the damage criterion *f_nCL_* = 2 was recorded in the forging rolled with flat tools. The use of concave tools has a clear effect on reducing the value of the damage criterion. An increase in the radius *R* of the concave tool from a value of 2500 mm to 5000 mm results in a gradual decrease in the damage function value. The smallest value of the damage function was recorded for a tool with a radius *R* = 5000 mm and was *f_nCL_* = 1.6. Increasing the value of radius *R* up to 6250 mm caused the value of the damage function to begin to increase, which can be seen at the locations marked ‘zone *A*’ in Figure 16. Increasing the radius *R* causes the concave tool to begin to resemble a flat tool, with the result that the degree of girding of the forging during rolling is reduced. When the area of the forging being banded by the tool is reduced, the effectiveness of blocking the material from over-ovalisation is also reduced. It is worth noting that the observed differences in damage function values for concave tools with radii *R* = 3750 mm, *R* = 5000 mm and *R* = 6250 mm are small. The areas of the differences in the damage function values are also small.

### 5.3. Analysis of Results and Discussion

**Figure 17:**
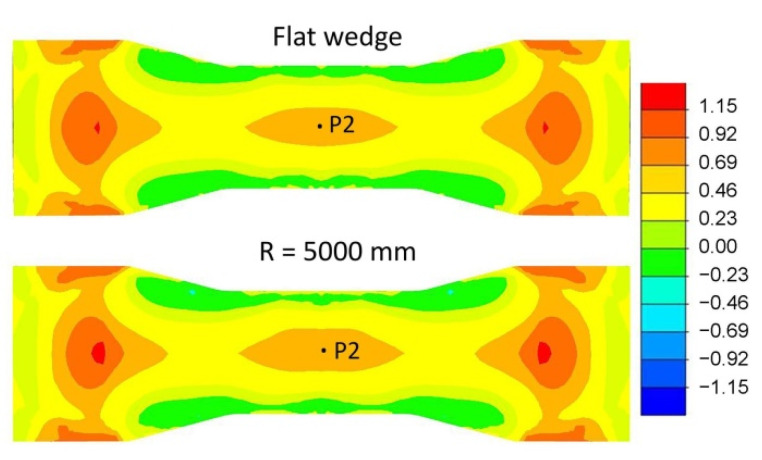
Distribution of the stress-state indicator values *σ*_1_*/σ_i_* for the least and most favourable conditions.

Figure 17 shows the distribution of the stress-state indicator *σ*_1_*/σ_i_* with an identical process step of 50%. The results in Figure 17 are restricted to the least favourable case of flat-wedge rolling and the most favourable case of concave-wedge rolling with a radius *R* = 5000 mm. From the distributions shown, it can be seen that changing the tool geometry does not cause significant changes in the distribution of the stress-state indicator value *σ*_1_*/σ_i_*.

**Figure 18:**
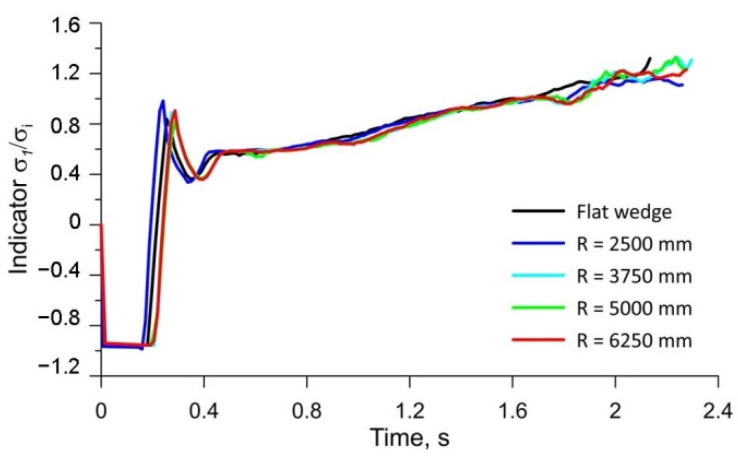
Variation in the stress-state indicator *σ*_1_*/σ_i_* at point P2.

Figure 18 shows the variation in the stress-state indicator *σ*_1_*/σ_i_* throughout the rolling process for point P2 (Figure 17). The variation curves of the stress-state indicator *σ*_1_*/σ_i_* are identical for all tool types analysed. The values of the stress-state indicator *σ*_1_*/σ_i_* are also identical for all tool types.

**Figure 19:**
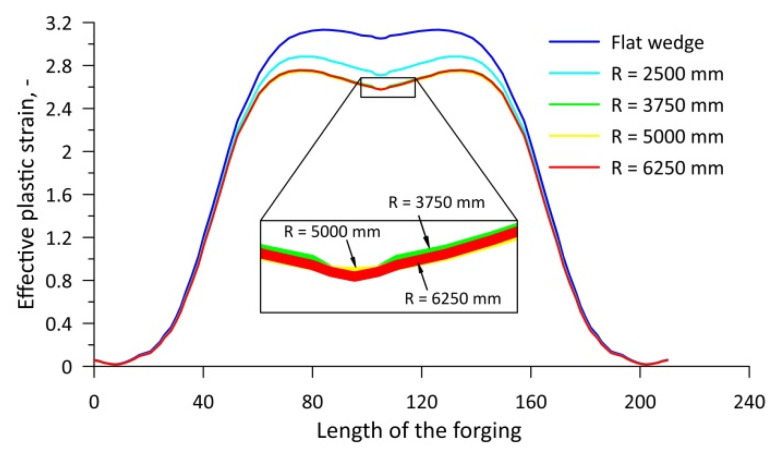
Distribution of the numerical values of the effective plastic strain along the central axis of the forgings.

Figure 19 shows the course of the variation in effective plastic strain values along the central axis of the rolled forgings. From the distribution shown, it is obvious that the use of concave tools reduces the value of effective plastic strain in the forging compared to a forging rolled with flat tools. An increase in the value of the radius *R* of concave tools up to a value of 3750 mm results in a reduction in the value of effective plastic strain. A subsequent increase in the value of the radius of the concave wedge (*R* = 5000 mm and *R* = 6250 mm) causes no change in the values of effective plastic strain. The lack of an influence of tool geometry on the value of the stress-state indicator *σ*_1_*/σ_i_* (Figure 18) confirms that the value of the normalised Cockcroft–Latham damage criterion is mainly dependent on the strain state. This observation is confirmed by the fact that the nature of the effective plastic strain distribution is reflected in the distribution of the value of the normalised damage criterion shown in Figure 16.

**Figure 20:**
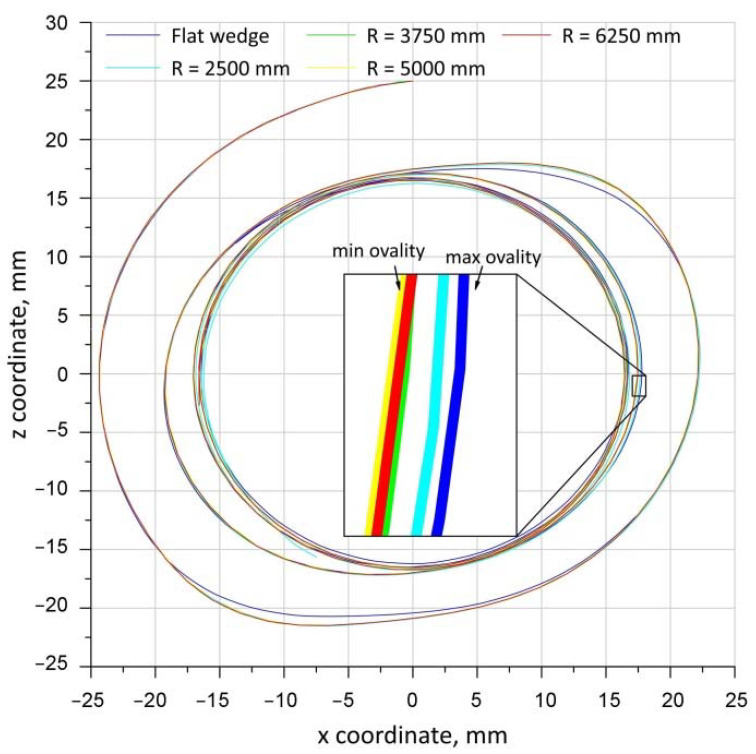
Trajectories of movement of point P1 for different wedge-tool geometries.

Figure 20 shows the trajectories of the P1 point for concave wedges. The initial and final locations of the P1 point for concave wedges were identical to those in the analysis of trajectories with variable rolling temperatures (Figure 11). The obtained trajectories of the movement of the P1 point for different wedge-tool geometries are shown in Figure 20. The presented trajectories show that the highest ovality of the cross-section of the forging occurs when rolling with a flat tool. The smallest ovalisation of the forging cross-section during rolling occurred for concave tools with radii *R* = 3750 mm, *R* = 5000 mm and *R* = 6250 mm. For the concave tool with a radius of *R* = 2500 mm, an intermediate value of the ovalisation of the forging cross-section was recorded. The recorded ovalisation of the cross-section of the forgings is reflected in the values of the effective plastic strain shown in Figure 19. Greater ovalisation of the cross-section causes an increase in redundant strain, which is generated by lengthening the forming path needed to remove the ovalisation of the cross-section. It can therefore be concluded that the amount of ovalisation of the cross-section of the forging during rolling determines the propensity of the material to fracture by influencing the effective plastic strain values.

## 6. Conclusions

This paper presents the results of a study on the fracture of material forming in the cross-wedge rolling process. In the first part of the study, it is shown that the process realisation temperature and the temperature-dependent ovalisation of the cross-section of the rolled forging have a significant influence on the size of the central crack in the forging. In the second part of the article, cross-wedge rolling is proposed with concave wedges, whose geometry makes it possible to limit the ovalisation of the cross-section of the forging during rolling. Numerical simulations of rolling with concave wedges with various degrees of concavity determined by the radius of the curvature of the wedge were carried out. The following final conclusions were drawn based on the research carried out:

- The size of the resulting material crack is dependent on the size of the ovalisation of the cross-section of the CWR-rolled forging;
- As the ovality of the forging cross-section increases during the CWR process, the value of the normalised Cockcroft–Latham damage criterion increases, and the size of the resulting crack increases;
- The use of concave tools reduces the size of the ovalisation of the cross-section of the forging in the CWR process and reduces the value of the normalised Cockcroft–Latham damage criterion;
- The smallest value of the normalised Cockcroft–Latham damage criterion was recorded for a concave tool with a radius of curvature *R* = 5000 mm.

## Figures and Tables

**Table 1:** Ovalisation sizes of the cross-section of forgings.

Temperature (°C)	d_max_ (mm)	d_min_ (mm)	Δd = d_max_ − d_min_ (mm)
950	25.37	21.61	3.76
1050	25.17	21.81	3.36
1150	24.75	22.03	2.72
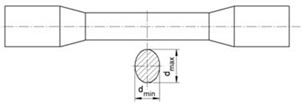			

## Data Availability

Data is contained within the article.
